# Rational Design and Synthesis of Diverse Pyrimidine Molecules Bearing Sulfonamide Moiety as Novel ERK Inhibitors

**DOI:** 10.3390/ijms20225592

**Published:** 2019-11-08

**Authors:** Ahmed H. Halawa, Areej A. Eskandrani, Walid E. Elgammal, Saber M. Hassan, Ahmed H. Hassan, Hassan Y. Ebrahim, Ahmed B. M. Mehany, Ahmed M. El-Agrody, Rawda M. Okasha

**Affiliations:** 1 Chemistry Department, Faculty of Science, Al-Azhar University, Nasr City, Cairo 11284, Egypt; ahmedhalawa_79@yahoo.com (A.H.H.); walid.elgammal85@gmail.com (W.E.E.); saber_js@yahoo.com (S.M.H.); ahmed_hassan7979@hotmail.com (A.H.H.); elagrody_am@yahoo.com (A.M.E.-A.); 2 Chemistry department, Faculty of Science, Taibah University, Medina 30002, Saudi Arabia; a.eskandrani@gmail.com; 3 Chemistry Department, Faculty of Science, Jazan University, Jazan 45142, Saudi Arabia; 4 Pharmacognosy Department, Faculty of Pharmacy, Helwan University, Cairo 11795, Egypt; hebrahim@pharm.helwan.edu.eg; 5 Zoology Department, Faculty of Science, Al-Azhar University, Nasr City, Cairo 11284, Egypt; abelal_81@yahoo.com

**Keywords:** extracellular signal-regulated kinases (ERK), anti-proliferative, rational design, sulfonamides, pyrazole, pyrimidine

## Abstract

Protein kinases orchestrate diverse cellular functions; however, their dysregulation is linked to metabolic dysfunctions, associated with many diseases, including cancer. Mitogen-Activated Protein (MAP) kinase is a notoriously oncogenic signaling pathway in human malignancies, where the extracellular signal-regulated kinases (ERK1/2) are focal serine/threonine kinases in the MAP kinase module with numerous cytosolic and nuclear mitogenic effector proteins. Subsequently, hampering the ERK kinase activity by small molecule inhibitors is a robust strategy to control the malignancies with aberrant MAP kinase signaling cascades. Consequently, new heterocyclic compounds, containing a sulfonamide moiety, were rationally designed, aided by the molecular docking of the starting reactant 1-(4-((4-methylpiperidin-1-yl)sulfonyl)phenyl)ethan-1-one (**3**) at the ATP binding pocket of the ERK kinase domain, which was relying on the molecular extension tactic. The identities of the synthesized compounds (**4**–**33**) were proven by their spectral data and elemental analysis. The target compounds exhibited pronounced anti-proliferative activities against the MCF-7, HepG-2, and HCT-116 cancerous cell lines with potencies reaching a 2.96 μM for the most active compound (**22**). Moreover, compounds **5**, **9**, **10b**, **22**, and **28** displayed a significant G2/M phase arrest and induction of the apoptosis, which was confirmed by the cell cycle analysis and the flow cytometry. Thus, the molecular extension of a small fragment bounded at the ERK kinase domain is a valid tactic for the rational synthesis of the ERK inhibitors to control various human malignancies.

## 1. Introduction

Protein kinases perform central regulatory roles in cell biology, namely: cell growth, proliferation, survival, differentiation, and metabolic functions. However, their dysregulation mediates the pathogenesis of many disorders [1,2]. Accordingly, protein kinases are established as promising druggable targets for controlling hyperproliferative diseases, including human cancers.

The Mitogen-Activated Protein (MAP) kinase is a pivotal oncogenic module in many human malignancies, which transmits mitogenic extracellular signals to intracellular effector targets [3]. The MAP kinase signaling pathway is triggered by diverse transmembrane receptors as epidermal growth factor (EGFRs) and hepatocyte growth factor (HGFR) receptors. Upon activation via specific ligands, these cell surface receptors become autophosphorylated at the intracellular domains, which is proceeded by their function as a docking scaffold for downstream adaptor proteins as guanine nucleotide exchange factors (GEFs). The preceding factors mediate the activation of a small GTPases rat sarcoma (Ras), located in the inner leaflet of the cell membrane. The Ras-GTP stimulates the dimerization and activation of downstream protein-serine/threonine kinase Raf (rapidly accelerated fibrosarcoma). As a result, the Raf catalyzes the phosphorylation and activation of MEK1/2, which sequentially, invokes the catalysis of the phosphorylation of ERK1/2 (the extracellular signal-regulated kinases). ERK1/2 are serine/threonine kinases that are able to accelerate the response of the phosphorylation of numerous diverse cytosolic and nuclear mitogenic proteins, as RSK1 (ribosomal S6 kinase 1). Therefore, the dysregulation of the MAP kinase pathway, either through the overexpression and/or activation of the oncogenic extracellular receptors or the cytoplasmic downstream effectors-Raf, MEK, and ERK, would promote the uncontrolled proliferation characteristic of cancerous cells [4].

Due to the recurrent MAP kinase-dependent malignancies, a substantial effort has been dedicated to the discovery of small molecule ERK1/2 inhibitors. Through this vigor, it has led to the unearthing of approximately 35 small molecule ERK1/2 inhibitors in different phases of clinical trials [5]. Most of these inhibitors encompass nitrogen-containing heterocyclic scaffolds with amide functionality that connect the heterocyclic ring system.

Disparate from the preceding reports, our report administers the incorporation of the sulfonamide moiety. Sulfonamides comprise of a remarkable category of medicinally efficient derivatives and possess several biological activities, such as antimicrobial [6,7,8,9,10], antiviral [11], insulin-releasing [12,13,14], anti-carbonic anhydrase [15,16,17], hypoglycemic [18], anti-inflammatory [19], antiglaucoma [20,21], anti-tumor [22,23,24] activities and effects. Additionally, pyrazole and pyrimidine nuclei are pharmacophoric scaffolds and exemplify a group of heterocyclic compounds with a large range of biological applications, including: antimicrobial [25,26,27,28,29,30,31,32,33,34,35,36], anticancer [37,38,39,40,41,42,43,44,45,46], analgesic and anti-inflammatory [47,48], antileishmanial [49,50,51], and antimalarial activities [50,51,52].

Herein, we register a molecular extension strategy of 1-(4-((4-methylpiperidin-1-yl)sulfonyl)-phenyl)ethan-1-one, aided by the molecular docking at the ERK kinase domain. The reactivity of the acetyl group was employed as a starting point for the extension towards the core of the ATP binding site of the ERK kinase pocket. The diverse pyrazole, pyrimidine, triazolo [4,3-*a*]pyrimidine, pyrazolo[1,5-*a*]pyrimidine, and pyrido[2,3-*d*]pyrimidine derivatives were synthesized and presented excellent binding poses as well as interactions with critical amino acids at the kinase domain. Moreover, they exhibited significant anti-proliferative activities against three cancerous cell lines with potencies reaching low micromolar levels. The most active molecules were evaluated for their impacts on cell cycle distribution and induction of apoptosis in multiple cancerous cell lines.

## 2. Results and Discussion

### 2.1. Chemistry

**Scheme 1:**
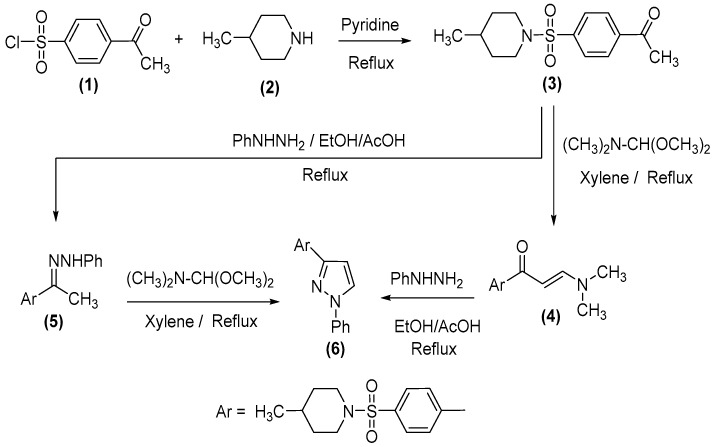
Synthesis of precursor **3** and derivatives **4**–**6**.

**Scheme 2:**
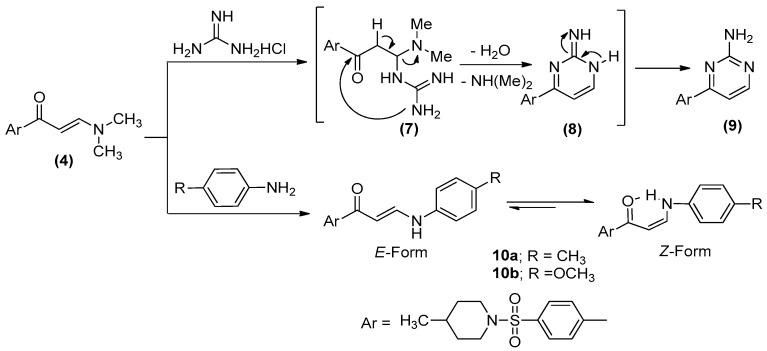
Synthesis of derivatives **9** and **10a**, **10b**.

**Scheme 3:**
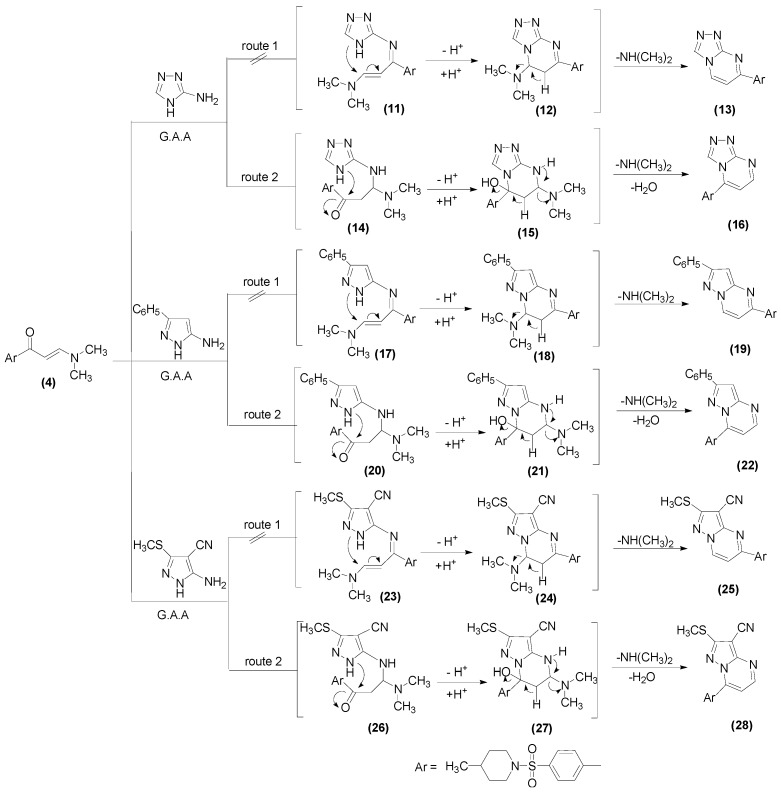
Synthesis of derivatives **13**, **16**, **19**, **22**, **25**, and **28**.

**Scheme 4:**
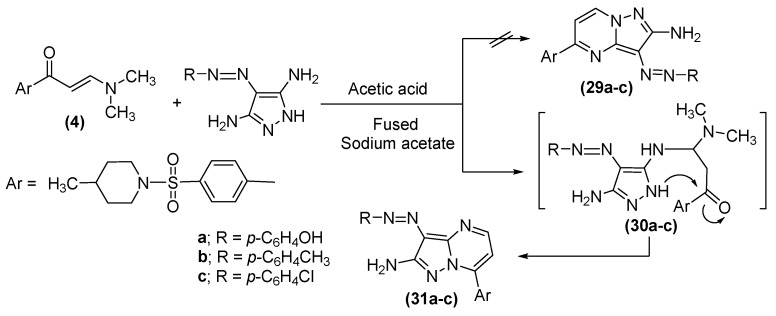
Synthesis of derivatives **31a–c**.

**Scheme 5:**
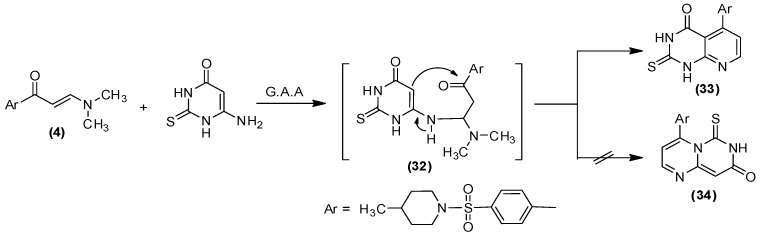
Synthesis of derivative **33**.

The chemistry employed for the preparation of the novel target molecules and the general synthetic pathways are illustrated in Scheme 1, Scheme 2, Scheme 3, Scheme 4 and Scheme 5. Through this work, we illustrate a proficient methodology for the production of several new heterocycles, containing a sulfonamide moiety, exploiting 1-(4-((4-methylpiperidin-1-yl)sulfonyl)phenyl)ethan-1-one (**3**) as a precursor. The aforementioned derivative was generated through the reaction of 4-acetylbenzenesulfonyl chloride (**1**) [53] with 4-methylpiperidine (**2**). The **c**ondensation of compound **3** with dimethylformamide-dimethylacetal (DMF-DMA) under reflux conditions in dry xylene afforded the corresponding enaminone (**4**) while the reaction of **3** with phenylhydrazine in an ethanol/acetic acid solution, delivered the phenylhydrazone derivative (**5**).

The reaction of the enaminone (**4**) with phenylhydrazine in the refluxing EtOH/AcOH offered the benzenesulfonyl-piperidine molecule (**6**). Alternatively, derivative **6** could be attained via the interaction of compound **5** with DMF-DMA in boiling xylene, as portrayed in Scheme 1. The structural composition and purity of the yielded molecules **4**–**6** were verified through their spectral data. The IR spectra of the molecules **3** and **4** validated the existence of the distinctive CO absorption bands at the *υ* 1690 cm^−1^, *υ* 1649 cm^−1^, and NH and at *υ* 3332 cm^−1^ for compound **5**.

On the other hand, the ^1^H NMR spectra of derivatives **3**–**6** divulged the singlet resonances of the acetyl, methyl, –N(Me)_2_, and pyrazol protons at δ 2.6, δ 2.3, δ 3.0, δ 3.2, δ 6.8, and δ 7.8 ppm, respectively. The ^13^C NMR spectra of derivatives **3** and **4** displayed signals, resonating at *δ* 26.9 and *δ* 45.1 ppm, which is attributable to the acetyl and –*n*(Me)_2_ carbons. Furthermore, enaminone (**4**) was assigned an *E*-configuration, established on its ^1^H NMR spectrum, which disclosed two singlet signals at δ 5.9 and δ 7.8 ppm that correspond to the two trans-olefinic protons. The coupling constant of the doublet signals for the olefinic protons equate to 16 Hz, which is associated with *E*-isomer. Furthermore, the MS spectra of compounds **3**–**6** provided the conclusive affirmation for their structures.

The interaction of enaminone (**4**) with guanidine hydrochloride in a refluxing ethanol/acetic acid atmosphere in the incidence of anhydrous potassium carbonate presented the sulfonyl pyrimidine derivative (**9**) while refluxing compound **4** with the principal aromatic amines, specifically: *p*-toluidine and *p*-anisidine, in an ethanolic/acetic acid mixture produced sulfonamide derivatives (**10a**, **10b**), Scheme 2.

The formation of **9** was presumed to ensue through the addition of the amino moiety of guanidine to an α,β-unsaturated group of enaminone **4** to generate the analogous acyclic non-isolable intermediate **7**, followed by the exhibition of the intramolecular cyclization and subsequent aromatization by the elimination of water and the dimethylamine compounds to deliver the concluding outcome **9**, Scheme 2. Meanwhile, the ^1^H NMR spectra of molecules **10a**, **10b** advocates that their demonstrated structure is *Z*-form, in which the coupling constant equates to 8 and 7.6 Hz, respectively. Additionally, the intramolecular hydrogen bonding afforded the stability of the *Z*-form, Scheme 2.

Enaminones formerly have been employed as prospective precursors for fused heterocyclic sets, when responding with heterocyclic amines [54,55,56,57]. Consequently, the action of enaminone (**4**) with several heterocyclic amines, such as 3-amino-1*H*-1,2,4-triazole, 5-phenyl-2*H*-pyrazol-3-ylamine, and 5-amino-3-methylthiopyrazole-4-carbonitrile in acetic acid resulted in new ring systems of triazolo[4,3-*a*]pyrimidine (**16**), pyrazolo[1,5-*a*]pyrimidine (**22**), and pyrazolo[1,5-*a*]pyrimidine-3-carbonitrile (**28**), respectively, Scheme 3.

In order to yield the molecules **16**, **22**, and **28**, two promising methods have been proposed to develop a credible mechanistic pathway, as illustrated in Scheme 3. Through the first course, the exocyclic amino moiety attacks the carbonyl group to afford the intermediates **11**, **17**, and **23**, which is pursued by the prior methodology of cyclization to afford the final products **13**, **19**, and **25**. Alternatively, the second route utilizes the Michael addition of the nucleophilic exocyclic amino moiety to the enaminone double bond, which allows the introduction of the intermediates **14**, **20**, and **26**, followed by dehydrated cyclization to produce **16**, **22**, and **28**, as exemplified in Scheme 3. The latter path has been unambiguously substantiated through the ^1^H NMR spectra, demonstrating two doublets in the regions *δ* 7.60–7.94 and *δ* 8.35–9.03 ppm, and whose coupling constant, *J* = 12–4.4 Hz, has been designated as a characteristic for the two vicinal *H*-5 and *H*-6 in the pyrimidine moieties [58,59].

In addition, the behavior of enaminone (**4**) towards the diazotized amino heterocyclic molecules was scrutinized. Subsequently, the reaction of **4** with 4-(4-(hydroxyl, methyl or chloro)phenylazo)-1*H*-pyrazole-3,5-diamine [60] in a refluxed acetic acid solution, in the presence of fused sodium acetate, generated the corresponding pyrazolo[1,5-*a*]pyrimidine derivatives (**31a–c**), as shown in Scheme 4.

The structural composition of **31a–c** was established on the foundation of their elemental analysis and spectral data, which will exclude the other possible structures (**29**). The IR spectra of compounds **31a–c** confirmed the absence of carbonyl absorption band as well as the appearance of the OH band at *υ* 3438 cm ^−1^ for compound **31a**. The ^1^H NMR spectra of derivatives **31a**, **b** exhibited a singlet at *δ* 9.9 ppm (exchangeable by D_2_O), due to the hydroxyl group; two doublet signals at δ 7.3 and 8.6 ppm, attributable to the pyrimidine *H*-5 and *H*-6, for compound **31a**; and a singlet resonance at δ 2.4 ppm, linked to the new methyl moiety, for compound **31b**. Moreover, the MS spectra of **31a–c** provided verification for the structural data of **31a–c**. Finally, the reaction of enaminone (**4**) with 6-Amino-2-thioxo-2,3-dihydro-1*H*-pyrimidine-4-one in acetic acid stemmed the synthesis of derivative (**33**), as displayed in Scheme 5.

In the above Scheme, compound (**33**) developed as a product of the cyclization of the intermediate **32**, which was preceded by the Michael addition procedure, rather than molecule (**34**). The identity of **33** was confirmed through its elemental analyses and spectral data. The observed ^1^H-NMR spectrum declared two singlet peaks at *δ* 12.65 and *δ* 13.24 ppm, assigned to the 2NH protons, which is only obtainable from isomer **33**, whereas the mass spectra revealed a peak at 416, comparable to the molecular ion value.

### 2.2. Biological Activity

#### In Vitro Anti-Proliferative Activity

**Figure 1:**
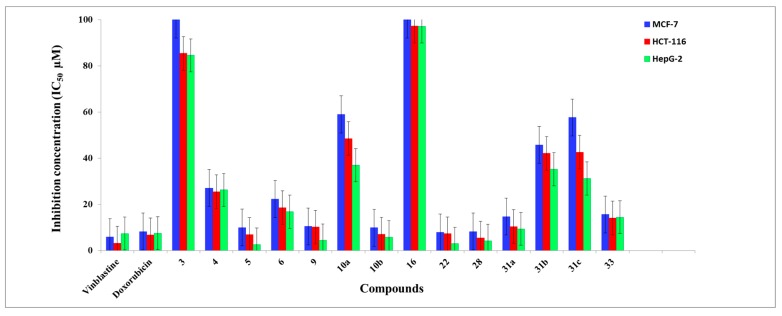
The IC_50_ values expressed in (µM) of the target compounds against the MCF-7, HCT-116, and HepG-2 tumor cells.

The anti-proliferative character of all the target derivatives **3**–**6**, **9**, **10**, **16**, **22**, **28**, **31**, and **33** were evaluated in vitro against three human tumor cell lines, mammary gland breast cancer (MCF-7), human colon cancer (HCT-116), and liver cancer (HepG-2), in comparison with Doxorubicin and Vinblastine as reference drugs, utilizing the standard sulphorhodamine B (SRB) assay [61]. The in vitro anti-proliferative examination was carried out under several concentrations, 0–100 μM, where the obtained data were expressed as proliferation inhibitory concentration (IC_50_) values, Figure 1 and Table 1.

The toxicity assay explicated that the novel compounds demonstrated superb to fair growth inhibitory features towards the screened cell lines. The assessment of the anti-proliferative behavior on the tumor cells revealed that HepG-2 administered the highest susceptibility, regarding the impact of the synthesized molecules. Meanwhile, derivatives **22** and **28** possess the most potency in evaluation against MCF-7, which are competitive and almost equipotent/equipotent with Doxorubicin, respectively. Furthermore, in comparison to the HCT-116 cell line, molecule **28** exhibited the highest potent behavior while molecules **5**, **10b**, and **22** were almost equipotent as Doxorubicin. Alternatively, the prepared molecules **5**, **22**, **28**, **9**, and **10b** presented a more significant impact of against HepG-2 in assessment against Vinblastine and Doxorubicin, whereas derivative 31a was almost equipotent as Vinblastine and Doxorubicin. The remaining molecules demonstrated modest cytotoxicity in evaluation to Vinblastine and Doxorubicin.

### 2.3. Molecular Docking and SAR Studies

**Figure 2:**
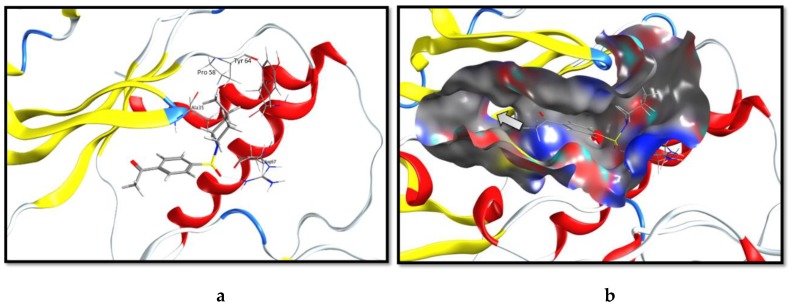
The virtual binding mode of compound **3** at the extracellular signal-regulated kinase 1 (ERK1) kinase domain. (**a**) The molecular interactions of compound **3** with amino acid residues at the binding pocket. (**b**) The surface representation of the binding mode of compound **3**, depicting the rational of the molecular extension (indicated by the arrow).

Compound **3**, (-(4-((4-methylpiperidin-1-yl)sulfonyl)phenyl)ethan-1-one, was docked at the ATP binding pocket of the ERK1 kinase domain (PDB code: 6MGD). As depicted in Figure 2a, this molecule was anchored at the kinase domain through a hydrogen bond with a side-chain guanido group of Arg 67. The 4-methylpyridinyl- moiety was imbedded in a hydrophobic sub-pocket created by amino acids Ala 35, Pro 58, and Try 64. Examining the binding pocket by the surface representation of the ligand binding site (Figure 2b) revealed that it was relatively twisted and elongated. The 4-methylpyridinyl- moiety was oriented towards the terminal of the pocket while the phenyl ethan-1-one moiety was directed inwards. This binding pose sparked a molecular extension hypothesis as to fill the unoccupied pocket with moieties that are able to create new molecular interactions which, consequently, improve the overall binding affinity.

The chemically reactive acetyl group of molecule **3** was employed as a precursor for its extension towards the deep end of the pocket. We adopted Scheme 1, Scheme 2, Scheme 3, Scheme 4 and Scheme 5 for synthesizing monocyclic and fused heterocyclic moieties, linked at the phenyl group’s para position for **3**. In the first Scheme, **3** was condensed with dimethylformamide-dimethylacetal (DMF-DMA) to afford (*E*)-3-(dimethylamino)-1-(4-((4-methylpiperidin-1-yl)sulfonyl)phenyl)prop-2-en-1-one (**4**). Subsequently, the anti-proliferative activity of the enaminone (**4**) was assessed in vitro against three cancerous lines (breast cancer ‘‘MCF-7’’, liver cancer “HepG-2”, and colon cancer “HCT-116”) and to the parent (**3**). Results divulged that the bioactivity of **4** was superior by more than 3-folds, reaching approximately 25 μM against HCT-116, in appraisal to 85 μM for **3** (Table 1). Grounded on these outcomes, a further extension of **4** was fashioned via the reaction of **3** with phenylhydrazine to allow 4-methyl-1-[4-(1-phenyl-1*H*-pyrazol-3-yl)benzenesulfonyl]piperidine (**6**). However, the aforesaid extension had not enhanced the bioactivity as expected against the exploited tumor cell lines. In an additional attempt, derivative **3** was arranged to react with phenylhydrazine to yield phenylhydrazone derivative (**5**). The anti-proliferative activity of **5** was significantly potentiated against the evaluated carcinogenic lines, attaining 2.5 μM in the HepG-2 cells.

**Figure 3:**
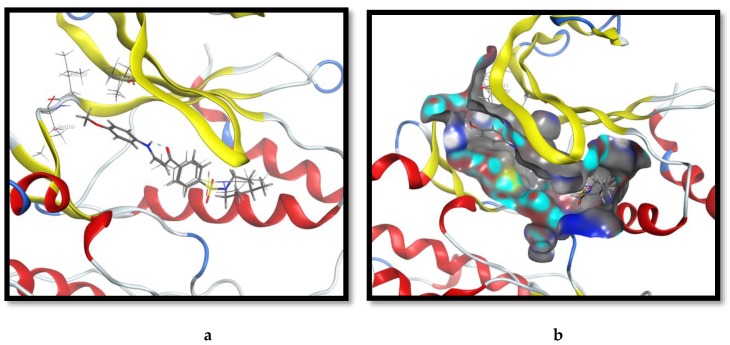
The virtual binding mode of compound **10b** at the ERK1 kinase domain. (**a**) The superposition of **10b** with **4** at the binding pocket, with the molecular interactions of **10b** with nonpolar amino acid residues. (**b**) The surface representation of the binding mode of **10b**, portraying the fitting of *p*-methoxy at a hydrophobic sub-pocket.

In Scheme 2, we reacted enaminone (**4**) with guanidine hydrochloride and diverse primary aromatic amines, *p*-toluidine and *p*-anisidine, to deliver4-[4-(4-methyl-piperidine-1-sulfonyl)-phenyl]pyrimidine-2-yl amine (**9**) and the 4-(3-((*p*-substituted amino) sulfonamide derivatives (**10a,b**), respectively. The pooled data from the anti-proliferative analyses disclosed that molecule **9** presented an improvement on bioactivity in comparison to molecule 4; however, it was comparable to the potency of the pyrazolo derivatives (**6**). Interestingly, **10a** and **10b** exhibited differential anti-proliferative potencies against the malignant cells: **10b** was relatively 6-folds higher than **10a** in bioactivity. In an endeavor to rational these disparate potencies, both derivatives were docked at the ATP binding site of the ERK kinase domain, and the binding poses were carefully examined. **10b** maintained the original binding pose of fragment **4** with an extension directed to the core of the pocket (Figure 3a). The *p*-OMe group was well-fitted into a hydrophobic sub-pocket, created by the side-chains of Ile 31, Ala 52, Leu 107, and Met 108. The extra oxygen-bridge in compound **10b** in assessment with 10a aided in the placement of the attached methyl group to the vicinity of a hydrophobic sub-pocket at the back of the binding site (Figure 3b).

**Figure 4:**
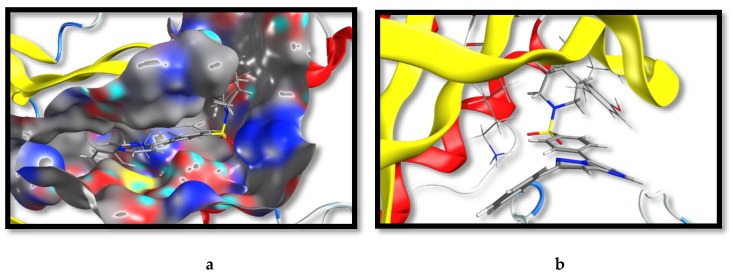
The virtual binding mode of compound **22** at the ERK1 kinase domain. (**a**) The surface representation, illustrating that **22** maintained the original binding pose as **3**, where **22** was extending deep in the pocket. (**b**) The interactions of **22** with amino acid residues at the kinase domain. Pyrazolo[1,5-*a*]pyrimidine exhibited a *π-π* interaction with the phenolic side-chain of Try36 and a cation-*π* interaction of the attached phenolic ring with the side-chain ammonium group of Lys54.

**Figure 5:**
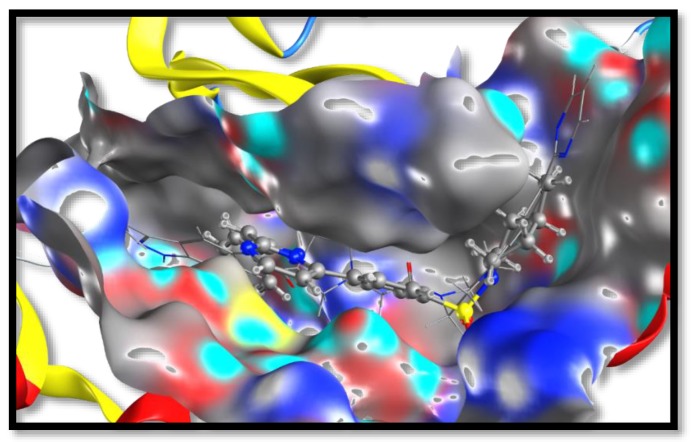
The overlay of the virtual binding mode of **22** (represented by ball and stick) and the X-ray binding mode of the co-crystalized ligand (represented by sticks) at the kinase domain. Both compounds revealed shallow U-shaped conformations with an X-ray co-crystalized ligand extending and occupying more space than **22**.

The third scheme demonstrates the aim of constructing fused azolopyrimidines ring systems through the reaction of enaminone (**4**) with different 2-amino di- and triazoles. The diverse products **16**, **22**, and **28** were appraised for their anti-proliferative potencies against the screened cell lines. Intriguingly, the bioactive behavior of **16** almost vanished, extending to approximately 100 μM in all cancerous cells. This is a valid indication that the fused triazolopyrimidine ring system is not an optimal scaffold for the molecular extension strategy. On the contrary, the pyrazolo[1,5-*a*]pyrimidine ring system represented by compounds **22** and **28** illustrated a significant development in the anti-proliferative potencies. Among all the synthesized molecules, compound **22** presented the highest potency in evaluation against HepG-2 cells with an IC_50_ of 2.96 μM, the most potent derivative overall. To virtually rational the higher potency of **22**, a molecular docking experiment was launched at the binding pocket of ERK. As portrayed in Figure 4a, derivative **22** maintained the original binding pose of precursor 3 (displayed by the overlay of **3** and **22**) with a molecular extension directed deeply to the pocket core. Additionally, the 2-pyrazolo[1,5-*a*]pyrimidine ring system exhibited a critical π–π interaction with the phenolic side-chain of Try36, located at the glycine rich ATP-phosphate binding loop (*p*-loop) within the small *n*-terminal lobe of the kinase domain. The assignment of the pyrazolo[1,5-*a*]pyrimidine scaffold to the attached phenolic ring enabled a cation –*π* interaction with the side-chain ammonium group of Lys 54 (Figure 4b). This amino acid residue is renowned in the anchorage of non-transferable α- and β-phosphates of ATP during the kinase reaction [62]. Certainly, these multiple molecular interactions of **22** with critical residues at the kinase domain would improve the overall binding affinity, which is translated as a noteworthy enhancement of the anti-proliferative potency. Subsequently, the virtual binding pose of **22** and X-ray binding pose of the co-crystalized ligand have been analyzed at the catalytic pocket (Figure 5). **22** was almost overlaid with the X-ray co-crystalized ligand and revealed a shallow U-shaped conformation, which substantiates the ERK inhibition hypothesis. However, the X-ray co-crystalized ligand was expanding in the pocket, occupying more space, in comparison to our product **22**. This could, at least, in part, justify the lower micromolar level of **22**.

By the fourth scheme, the enaminone **4** was reacted with different diazotized amino pyrazolo derivatives to yield the corresponding diazotized pyrazolo[1,5-*a*]pyrimidine derivatives (**31a–c**). Furthermore, all the furnished products were assessed in the in vitro anti-proliferative platform. The results indicated that derivative **31a**, which bears a *para* phenolic OH, exhibited the highest potency in this series with IC_50_, reaching 9.36 μM, against the HepG-2 line, in appraisal with 35 and 31 μM for **31b** “bearing *para* CH_3_” and **31c** “bearing *para* Cl”, respectively. This truly denoted the preference of a polar electron-donating group at the *para* position, which is capable of increasing the π cloud on the phenyl ring as well as forming hydrogen bonds with the nearby amino acids.

Lastly, constructing a fused 6 + 6 heterocyclic ring system was targeted through the reaction of enaminone **4** with the aminopyrimidine-4-one derivative to yield the corresponding pyrido[2–*d*]pyrimidin-4-one (**33**). The anti-proliferative assessment of **33** against the examined cancerous cell lines suggested a considerable improvement in the potency, relative to its precursor enaminone **4** (Table 1); however, **33** does not exceed the potency **22**. Thereby, a conclusion was formulated, in which the molecular extension of 1-(4-((4-methylpiperidin-1-yl)sulfonyl)phenyl)ethan-1-one (**3**) with the fused 5 + 6 heterocyclic ring systems, bearing an aromatic set, would be the optimal extension strategy towards cultivating the binding to the ERK kinase domain.

### 2.4. Analysis of Cell Cycle Distribution

The progressive cell cycle is considered a hallmark of cancer. Though normal cells have restrictions in cell cycle progression, allowing them to terminate cellular division under abnormal conditions (as metabolic imbalance or DNA damage), cancerous cells overcome checkpoints and proceed with cycle progression. Thus, the inhibition of the cell cycle is a promising therapeutic strategy for combating cancer, evidenced by the incidence of many drug candidates in numerous phases of clinical trials [5].

**Figure 6:**
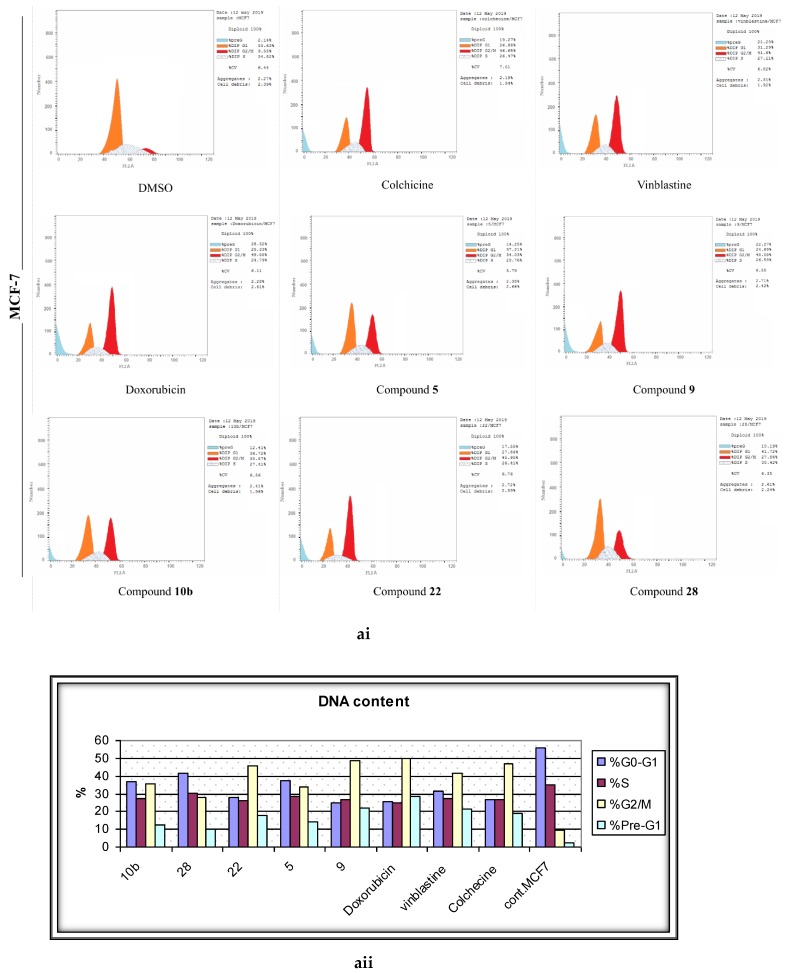

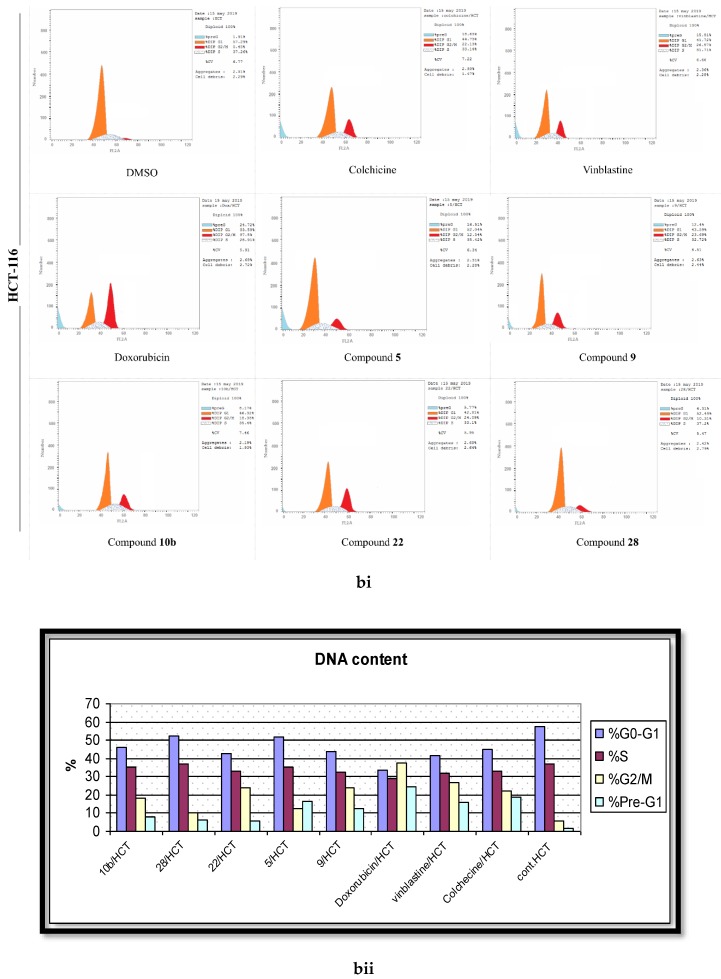

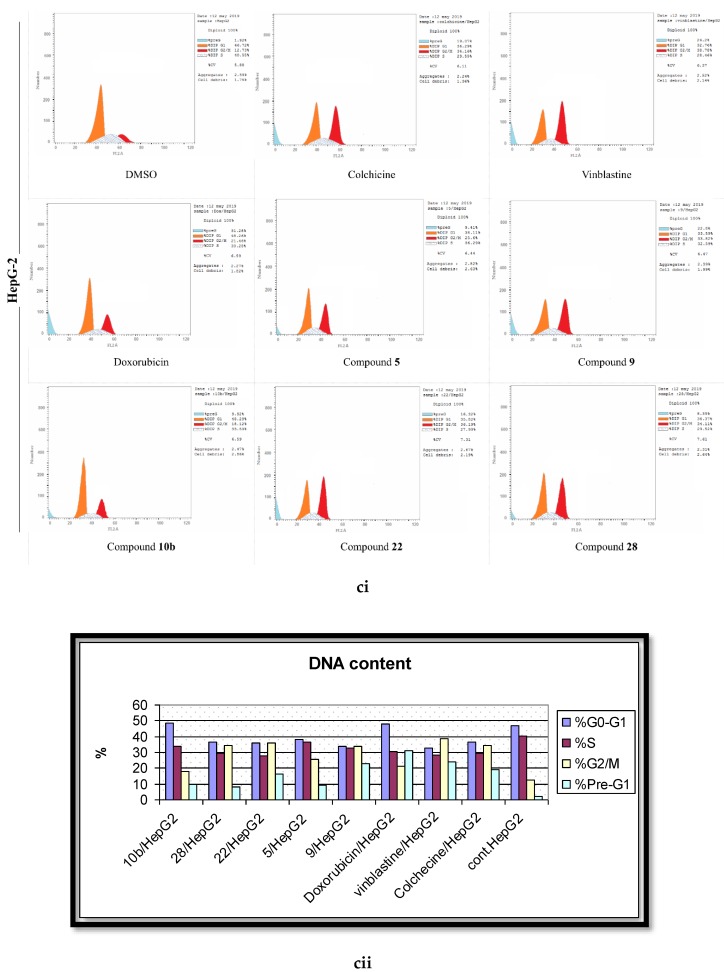
The effects of the DMSO, the standard anticancer drugs, and the synthesized compounds on the cell cycle distribution of the (**a**) MCF-7, (**b**) HCT, and (**c**) HepG-2 cancer cell lines. (**i**) FACS-mediated cell cycle analysis, (**ii**) A histogram shows the percentages of the DNA content in different cell cycle phases.

To gauge the dynamics of the cell cycle regulations during the remedy of cells by the active molecules (**5**, **9**, **10b**, **22**, **28**), the flow cytometric analyses were performed after the treatment of the malignant cell lines with the desired compounds at their corresponding IC_50_ values. The pooled results demonstrated a normal cell cycle distribution pattern in the DMSO-treated cells, where approximately 52% of the cells were in the G1 phase, 37% were in the S phase, 7% in the G2/M phase, and 2% were in the pre-G1 phase (Figure 6). In contrast, the standard positive control drugs (Colchicine, Vinblastine, and Doxorubicin) initiated a substantial G2/M phase arrest in the three cancerous lines. The phenylhydrazone derivative (**5**) stemmed 3.6, 2, and 2.3 fold surges in the cell percentage of the G2/M phase in MCF-7, HepG-2, and HCT-116 in assessment with the control cells. The 2-amino pyrimidine derivative 9 ensued a 39%, 21%, and 18% growth in cell population in the G2/M phase in MCF-7, HepG-2, and HCT-116, respectively. The enaminone derivative **10b** was relatively less pronounced than **9** towards the induction of the G2/M phase arrest; yet, the IC_50_ values of both compounds was comparable in their anti-proliferation assays. As anticipated, **22**, presenting the most potency, was the leading molecule for the generation of the G2/M phase arrest with MCF-7 being the most sensitive (45.95%) and HCT-116 being relatively resistant (24.09%). The other active (methylthio)pyrazolo[1,5,b]pyrimidine-3-carbonitrile derivative (**28**) established a relatively lesser potency in the stimulation of the G2/M cell population phase, when appraised against its counterpart from the same scheme (**22**). This highlights the focal role of the substituents, attached to the pyrazolo[1,5,b]pyrimidine scaffold on the cell cycle distribution.

### 2.5. Analysis of Cell Apoptosis

The aberrant activation of the ERK signaling pathway has been documented to overcome apoptosis, prompted by an extensive array of stimuli, such as tumor necrosis factor (TNF) [63], radiation [64], and chemotherapeutic agents [65]. The mechanism by which the ERK activation inhibits cell apoptosis is complicated, as it is depending on cell-type and other cellular regulatory effects. Consequently, the inhibition of the ERK activity by small molecules develop apoptotic induction. A recognized element of apoptotic cell death is the externalization of the phosphatidylserine (PS) residues from the plasma membrane’s internal leaflet to the external ones (known as the membrane lipid scrambling), thereby, facilitating the phagocytic recognition, engulfment, and destruction of the formed apoptotic bodies. Annexin-V is a protein, which specializes in binding to the phosphatidylserine (PS) at the cell surface, and the recognition of the externalized PS (apoptotic cells as well), through the means of the flow cytometric assay, is acquired via the authorization of the fluorescent labelling of the Annexin-V. To accurately distinguish amongst cells undergoing early apoptosis and late apoptosis/necrosis, the Annexin-V is employed in conjunction with propidium iodide “PI” (a sensor of living/dead cells, which gages membrane integrity).

**Figure 7:**
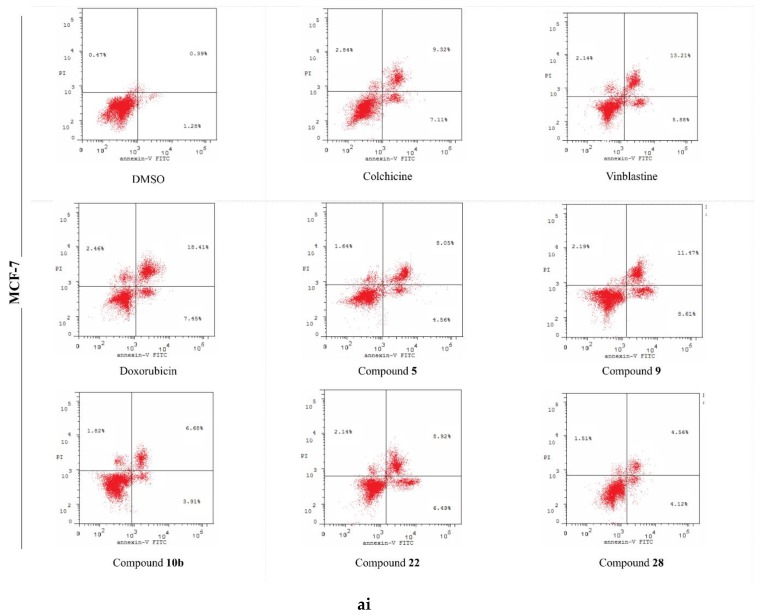

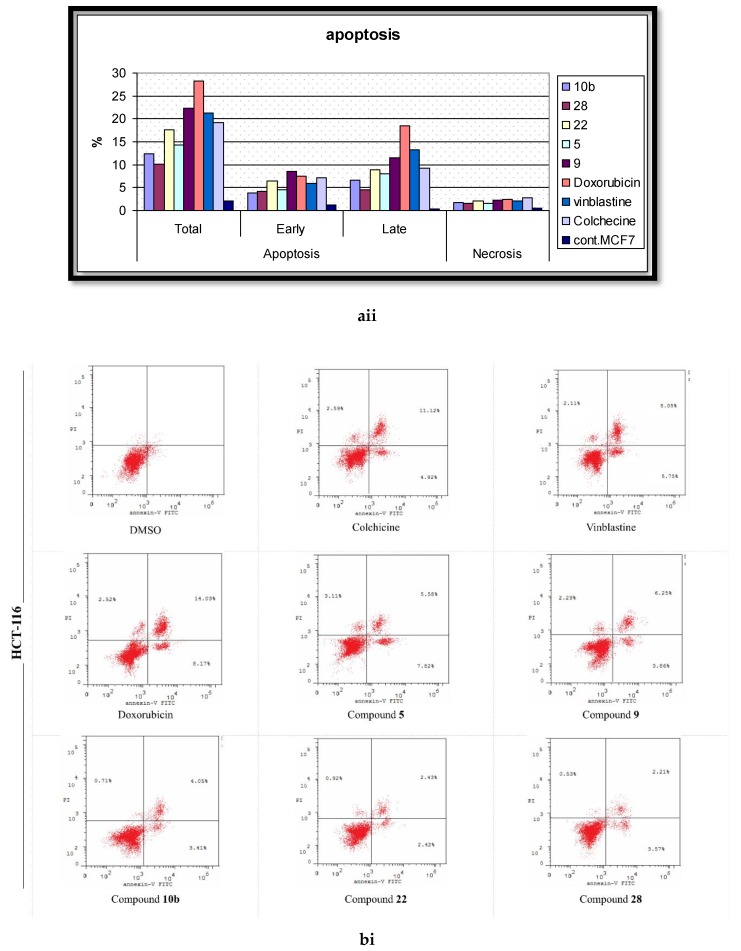

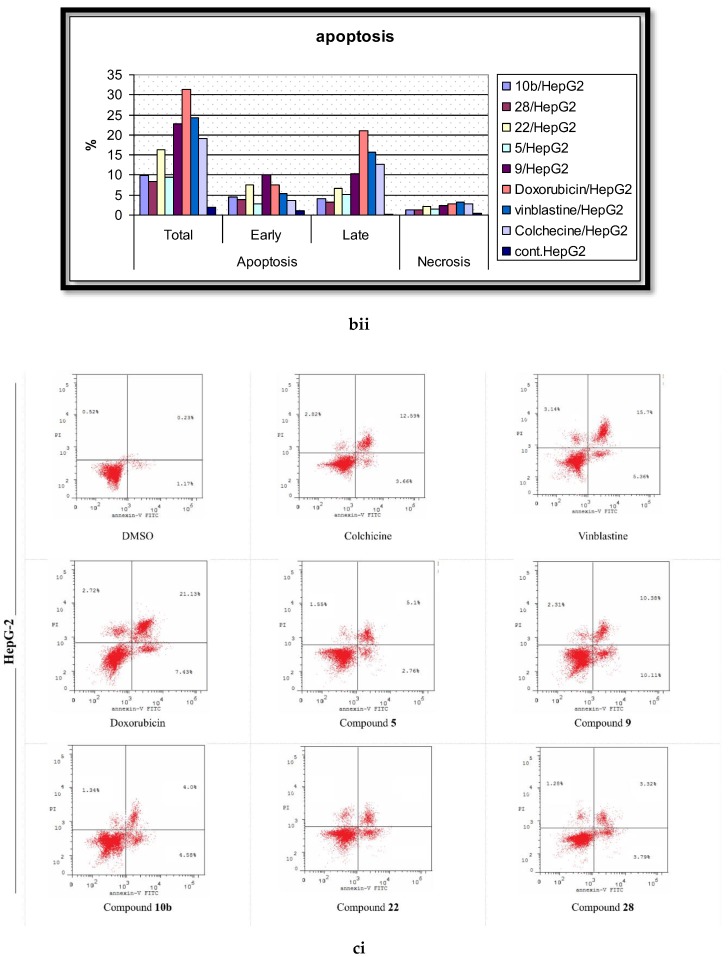

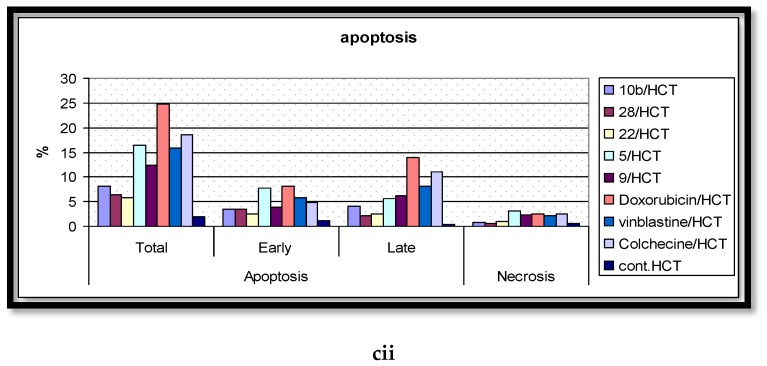
The effects of the DMSO, the standard anticancer drugs, and the synthesized compounds on the cell apoptosis of the (**a**) MCF-7, (**b**) HCT, and (**c**) HepG-2 cancer cell lines. (**i**) Scatter plots of Annexin-V FITC/PI cell staining. (**ii**) Histograms for the percentages of the cell apoptosis and necrosis.

In the present report, the Annexin V/PI double staining flow cytometric assay was exercised to assess the impact of the most active compounds on cell apoptosis. Cells were treated with DMSO (as a negative control); Colchicine, Vinblastine, Doxorubicin (as positive standard anticancer drugs); and the analyzed derivatives (**5**, **9**, **10b**, **22**, **28**) at their corresponding IC_50_ values. The outcomes are processed and summarized in Figure 7. The DMSO-treated cells disclosed the minimal percentage of total apoptosis, attaining 2.14% of the entire cell population in MCF-7 cells. Alternatively, the positive control-treated cells displayed significant elevated percentages of total apoptotic cell, up to 31.28, for the HepG-2 cells, remedied with Doxorubicin. Molecule **5** was the most effective against the MCF-7 line with a 14.25% apoptosis induction while being the least effective against HepG-2 with only 9.41%. The 2-amino pyrimidine derivative **9** stimulated higher comparable percentages of cell apoptosis in both the MCF-7 and HepG-2 cells with the production of minimal percentages for the HCT cells. Intriguingly, 9 was verified as the highest active molecule in the cell apoptosis evaluation; however, this case was not witnessed in the anti-proliferative assay. The deduction that can be constructed, established on these results, is that derivative **9** centers on targeting a potential apoptotic pathway rather than the ERK-mediated signaling cascade. Furthermore, compound **10b** was most effective against the MCF-7 cells, causing a 12.41% initiation of cell apoptosis with its least exhibition of 8.17% for HCT-116. The most potent anti-proliferative derivative (**22**) demonstrated extensive sensitivity: a 17.55% apoptosis induction (highest) for MCF-7, a moderate stimulation of cell apoptosis for HepG-2, and a 5.77% generation of apoptotic cell death (lowest) for HCT-116. Lastly, the analyzed molecule **28** illustrated a relatively lesser induction of cell apoptosis in assessment with **22**; however, both compounds possess the same imidazole[1,5,*a*]pyrimidine scaffold in the extension site. The aforementioned data suggests that the function groups, attached to the scaffold, performs a pivotal role in molecular interactions with biological targets.

## 3. Materials and Methods

### 3.1. General Methods

All melting points, measured with a SMP50 Digital APP (Bibby Scientific, Staffordshire, UK) 120/230V, are uncorrected. The IR spectra (KBr, *υ* cm^−1^) were recorded on the CARY 630 FT-IR spectrometer (Agilent, Santa Clara, CA, USA). The pre-coated silica gel plates (silica gel 0.25 mm, 60 G F 254; Merck, Germany) were employed for thin layer chromatography. The ^1^H/^13^C NMR (400/101 MHz) spectra were measured in (DMSO-d_6_) or (CDCl_3_) on a bruker NMR spectrometer (Bruker, Billerica, MA, USA). The mass spectra were attained through a GC Ms-QP 1000 EX mass spectrometer (Shimadzu, Kyoto, Japan) at 70 eV. The elemental analyses were executed on a Carlo Erba 1108 Elemental Analyzer (Heraeus, Hanau, Germany) at the Micro analytical Research Center, Faculty of Science, Cairo University (Cairo, Egypt). All of the compounds were within ± 0.4% of the theoretical values.

#### 3.1.1. 1-[4-(4-Methyl-piperidine-1-sulfonyl)phenyl]ethanone (**3**)

A mixture of 4-acetylbenzensulfonyl chloride (**1**) (0.01 mol), 4-methyl piperidine (**2**) (0.01 mol), and ether (50 mL) in the presence of pyridine (0.5 mL), as a catalyst, was stirred for 3 h at room temperature. The resulting solid was filtered, washed with dilute hydrochloric acid (37%), and recrystallized from ethanol to afford compound **3**. The physical and spectral data of compound **3** was as illustrated:

Colorless crystals; yield 65%; m.*p*. 120–122 °C; IR (KBr) *υ* (cm^−1^): 3075 (Ar–CH), 2928 (Ali–CH), 1690 (C=O), 1338, 1163 (SO_2_); ^1^H–NMR (400 MHz, CDCl_3_) *δ*: 0.9 (d, 3H, CH_3_), 1.3 (br s, 2H, CH_2_), 1.6 (br s, 2H, CH_2_), 1.8 (br s, 1H, CH), 2.3 (t, 2H, CH_2_–N–CH_2_), 2.6 (s, 3H, CH_3_CO), 3.7 (t, 2H, CH_2_–N–CH_2_), 7.9 (dd, 1H, *J* = 9.2, 4.4 Hz, AB–Ar–H), 8.1 (dd, 1H, *J* = 9.2, 4.8 Hz, AB–Ar–H); ^13^C-NMR (101 MHz, DMSO-*d_6_*) *δ*: 21.4, 26.9, 30.1, 30.1, 33.3, 46.4, 46.4, 127.9, 127.9, 128.8, 128.8, 140.0, 140.5, 196.9; MS *m*/*z* (%): 281 (M^+^, 30.2); Anal. Calcd for C_14_H_19_NO_3_S (281.37): C, 59.76; H, 6.81; N, 4.98; O; Found: C, 59.68; H, 6.72; N, 4.90%.

#### 3.1.2. (*E*)-3-(Dimethylamino)-1-(4-((4-methylpiperidin-1-yl)sulfonyl)phenyl)prop-2-en-1-one (**4**)

A mixture of 1-[4-((4-methylpiperidin-1-yl)sulfonyl)phenyl]ethenone (**3**) (0.01 mol) and dimethylformamide-dimethylacetal (DMF–DMA) (0.01 mol) in dry xylene (30 mL) was heated under reflux conditions for 5 h. The isolated solid was filtered off, washed with ethanol, and recrystallized from ethanol/benzene to yield molecule 4. The physical and spectral data of compounds 4 was as displayed:

Orange crystals; yield 92%; m.*p*. 212–214 °C; IR (KBr) *υ* (cm^−1^): 3050 (Ar–CH), 2927 (Ali–CH), 1649 (C=O), 1335, 1159 (SO_2_); ^1^H-NMR (400 MHz, DMSO-*d_6_*) *δ*: 0.9 (d, 3H, CH_3_), 1.1 (br s, 2H, CH_2_), 1.3 (br s, 1H, CH), 1.7 (br s, 2H, CH_2_), 2.2 (t, 2H, CH_2_–N–CH_2_), 3.0, 3.2 (2s, 6H, NMe_2_), 3.6 (t, 2H, CH_2_–N–CH_2_), 5.9, 7.8 (dd, 2H, *J* = 16 Hz, olefinic CH=CH), 7.8 (dd, 1H, *J* = 8 Hz, AB–Ar–H), 8.1 (dd, 1H, *J* = 8, 1.2 Hz, AB–Ar–H); ^13^C-NMR (101 MHz, DMSO-*d_6_*) *δ*: 21.8, 29.7, 29.7, 33.3, 37.7, 45.1, 46.5, 46.5, 91. 5, 127.8, 128.2, 128.4, 129.5, 137.6, 144.5, 155.5, 184.7; MS *m*/*z* (%): 336 (M^+^, 35.51); Anal. Calcd for C_17_H_24_N_2_O_3_S (336.45): C, 60.69; H, 7.19; N, 8.33; O; Found: C, 60.61; H, 7.10; N, 8.26%.

#### 3.1.3. (*E*)-4-Methyl-1-((4-(1-(2-phenylhydrazineylidene)ethyl)phenyl)sulfonyl)piperidine (**5**)

A mixture of 3 (0.01 mol) and phenylhydrazine (0.01 mol) in an ethanol/acetic acid solution (40 mL) (1:1) was refluxed for 4 h, during which a crystalline solid was separated. The separated solid was filtered off, washed with ethanol, and recrystallized from ethanol to produce compound 5. The physical and spectral data of derivative 5 was as follows:

Orange crystals; yield 91%; m.*p*. 176–178 °C; IR (KBr) *υ* (cm^−1^): 3332 (NH), 3056 (Ar–CH), 2927 (Ali-CH), 1649 (C=O), 1334, 1163 (SO_2_); ^1^H-NMR (400 MHz, DMSO-*d_6_*) *δ*: 0.9 (d, 3H, CH_3_), 1.2 (br s, 2H, CH_2_), 1.6 (br s, 1H, CH), 1.7 (br s, 2H, CH_2_), 2.2 (t, 2H, CH_2_–N–CH_2_), 2.3 (s, 3H, CH_3_C=N), 3.6 (t, 2H, CH_2_–N–CH_2_), 6.8–7.3 (m, 5H, phenyl ring), 7.7 (dd, 1H, *J* = 8 Hz, AB–Ar–H), 8.0 (dd, 1H, *J* = 8 Hz, AB–Ar–H), 9.6 (s, 1H, NH); MS *m*/*z* (%): 371 (M^+^, 48.51); Anal. Calcd for C_20_H_25_N_3_O_2_S (371.50): C, 64.66; H, 6.78; N, 11.31; O; Found: C, 64.72; H, 6.83; N, 11.37%.

#### 3.1.4. 4-Methyl-1-[4-(1-phenyl-1*H*-pyrazol-3-yl)benzenesulfonyl]piperidine (**6**)

Procedure (a): A mixture of enaminone (**4**) (0.01 mol) and phenylhydrazine (0.01 mol) in a solution of ethanol/acetic acid (40 mL) (1:1) was refluxed for 3 h. Upon cooling, the solid, which formed, was recrystallized from ethanol to generate compound **6**.

Procedure (b)*:* A solution of *(E)-4-methyl-1-((4-(1-(2-phenylhydrazineylidene)ethyl)phenyl) sulfonyl)piperidine* (**5**) (0.01 mol) in dry xylene (30 mL) and dimethylformamide-dimethylacetal (DMF-DMA) (0.01 mol) was refluxed for 5 h. Pursuing the same preceding methodology, molecule **6** was formed (m.*p*. and mixed m.*p*.). The physical and spectral data of compound 6 was as presented:

Pale yellow crystals; yield 86%; m.*p*. 140–142 °C; IR (KBr) *υ* (cm^−1^): 3068 (Ar–CH), 2927 (Ali–CH), 1338, 1163 (SO_2_); ^1^H-NMR (400 MHz, DMSO-*d_6_*) *δ*: 0.9 (d, 3H, CH_3_), 1.1 (br s, 2H, CH_2_), 1.6 (br s, 1H, CH), 1.7 (br s, 2H, 2CH_2_), 2.2 (t, 2H, CH_2_–N–CH_2_), 3.6 (t, 2H, CH_2_–N–CH_2_), 6.8 (dd, 1H, *J* = 4 Hz, pyrazole ring), 7.3 (dd, 1H, *J* = 4, AB–Ar–H), 7.4–7.5 (m, 5H, phenyl ring), 7.7 (dd, 1H, *J* = 4, AB–Ar–H), 7.8 (dd, 1H, *J* = 4 Hz, pyrazole ring); MS *m*/*z* (%): 381 (M^+^, 100); Anal. Calcd for C_21_H_23_N_3_O_2_S (381.49): C, 66.12; H, 6.08; N, 11.01; O; Found: C, 66.05; H, 6.00; N, 10.92%.

#### 3.1.5. 4-[4-(4-Methyl-piperidine-1-sulfonyl)-phenyl]pyrimidin-2-ylamine (**9**)

A mixture of enaminone (**4**) (0.01 mol) and guanidine hydrochloride (0.01 mol) in ethanol/acetic acid (30 mL) and anhydrous potassium carbonate (2 gm) was inserted. The resulting mixture was refluxed for 6 h, allowed to cool in room temperature, and diluted with water (20 mL). The solid product formed was collected by filtration, washed with water, and recrystallized from ethanol to afford compound **9**. The physical and spectral data of compound 9 was as demonstrated:

Pale yellow crystals; yield 87%; m.*p*. 208–210 °C; IR (KBr) *υ* (cm^−1^): 3487, 3330 (NH_2_), 3090 (Ar–CH), 2925 (Ali–CH), 1337, 1162 (SO_2_); ^1^H-NMR (400 MHz, DMSO-*d_6_*) *δ*: 0.9 (d, 3H, CH_3_), 1.2 (br s, 2H, CH_2_), 1.3 (br s, 1H, CH), 1.7 (br s, 2H, CH_2_), 2.3 (t, 2H, CH_2_–N–CH_2_), 3.7 (t, 2H, CH_2_–N–CH_2_), 6.8 (s, 2H, NH_2_), 7.2 (dd, 1H, *J* = 8 Hz, CH–pyrimidine ring), 7.8 (dd, 1H, *J* = 8 Hz, AB–Ar–H), 8.3 (dd, 1H, *J* = 8 Hz, AB–Ar–H), 8.4 (dd, 1H, *J* = 8 Hz, CH–pyrimidine ring); MS *m*/*z* (%): 332 (M^+^, 10.57); Anal. Calcd for C_16_H_20_N_4_O_2_S (332.42): C, 57.81; H, 6.06; N, 16.85; O; Found: C, 57.75; H, 5.98; N, 16.78%.

#### 3.1.6. General Procedure for Preparation of (**10a**, **b**)

A mixture of enaminone (4) (0.01 mol) and the primary aromatic amines: (*p*-toluidine and *p*-anisidine) (0.01 mol) in a solution of ethanol/acetic acid (40 mL) (1:1) was refluxed for 3 h, where a crystalline solid was separated. The separated solid was filtered off, washed with ethanol, and recrystallized from ethanol/benzene to yield compounds **10a**,**10b**. The physical and spectral data of compounds 10a, b was as shown:

#### 3.1.7. (*E*)-1-(4-((4-Methylpiperidin-1-yl)sulfonyl)phenyl)-3-(*p*-tolylamino)prop-2-en-1-one (**10a**)

Yellow crystals; yield 95%; m.*p*. 215–216 °C; IR (KBr) *υ* (cm^−1^): 3357 (NH), 3069 (Ar–CH), 2950 (Ali–CH), 1689 (C=O), 1338, 1162 (SO_2_); ^1^H-NMR (400 MHz, DMSO-*d_6_*) *δ*: 0.9 (d, 3H, CH_3_), 1.2 (br s, 2H, CH_2_), 1.3 (br s, 1H, CH), 1.7 (br s, 2H, CH_2_), 2.3 (t, 2H, CH_2_–N–CH_2_), 2.3 (s, 3H, CH_3_), 3.7 (t, 2H, CH_2_–N–CH_2_), 6.1 (d, 1H, *J* = 8 Hz, COCH), 7.1 (m, 1H, CH-NH), 7.2 (dd, 1H, *J* = 8 Hz, AB–Ar–H), 7.3 (dd, 1H, *J* = 8 Hz, AB–Ar–H), 7.8 (dd, 1H, *J* = 8 Hz, AB–Ar–H), 8.2 (dd, 1H, *J* = 8 Hz, AB–Ar–H), 12.2 (d, 1H, *J* = 12 Hz, NH); MS *m*/*z* (%): 398 (M^+^, 76.14); Anal. Calcd for C_22_H_26_N_2_O_3_S (398.52): C, 66.31; H, 6.58; N, 7.03; O; Found: C, 66.24; H, 6.51; N, 6.96%.

#### 3.1.8. (*E*)-3-((4-Methoxyphenyl)amino)-1-(4-((4-methylpiperidin-1-yl)sulfon-yl)phenyl)prop-2-en-1-one (**10b**)

Yellow crystals; yield 89%; m.*p*. 188–190 °C; IR (KBr) *υ* (cm^−1^): 3357 (NH), 3069 (Ar–CH), 2927 (Ali–CH), 1689 (C=O), 1338, 1162 (SO_2_); ^1^H-NMR (400 MHz, DMSO-*d_6_*) *δ*: 0.9 (d, 3H, CH_3_), 1.2 (br s, 2H, CH_2_), 1.3 (br s, 1H, CH), 1.7 (br s, 2H, CH_2_), 2.3 (t, 2H, CH_2_–N–CH_2_), 3.7 (t, 2H, CH_2_–N–CH_2_), 3.8 (s, 3H, OCH_3_), 6.1 (d, 1H, *J* = 7.6 Hz, COCH), 7.2 (m, 1H, CH–NH), 7.0 (dd, 1H, *J* = 8 Hz, AB–Ar–H), 7.4 (dd, 1H, *J* = 8 Hz, AB–Ar–H), 7.8 (dd, 1H, *J* = 8 Hz, AB–Ar–H), 8.2 (dd, 1H, *J* = 8 Hz, AB–Ar–H), 12.2 (d, 1H, *J* = 12 Hz, NH); ^13^C-NMR (101 MHz, DMSO-*d_6_*) *δ*: 21.8, 29.7, 29.7, 33.3, 46.5, 46.5, 55.8, 92.9, 115.4, 118.6, 128.1, 128.1, 128.3, 128.4, 133.6, 138.3, 142.8, 148.19, 156.6, 187.5; MS *m*/*z* (%): 414 (M^+^, 86.28); Anal. Calcd for C_22_H_26_N_2_O_4_S (414.52): C, 63.75; H, 6.32; N, 6.76; O; Found: C, 63.67; H, 6.25; N, 6.69%.

#### 3.1.9. General Procedure for Preparation of Compounds **16**, **22**, **28**

A mixture of enaminone (**4**) (0.01 mol) and 3-amino-1*H*-1,2,4-triazole or 5-phenyl-2*H*-pyrazol-3-ylamine or 5-amino-3-methylthiopyrazole-4-carbonitrile (0.01 mol) in acetic acid (30 mL) was refluxed for 3 h. The solvent was removed by the distillation under reduced pressure, and the resultant was left to cool. The solid precipitate was collected by filtration and recrystallized from ethanol and benzene, methanol and benzene, or ethanol to yield compounds **16**, **22**, and **28**, respectively. The physical and spectral data of compounds 1**6**, **22**, **28** were as follows:

#### 3.1.10. 5-[4-(4-Methyl-piperidine-1-sulfonyl)phenyl][1,2,4]triazolo[4,3-*a*]pyrimidine (16)

Colorless crystals; yield 80%; m.*p*. 209–210 °C; IR (KBr) *υ* (cm^−1^): 3070 (Ar–CH), 2924 (Ali–CH), 1340, 1168 (SO_2_); ^1^H-NMR (400 MHz, DMSO-*d_6_*) *δ*: 0.9 (d, 3H, CH_3_), 1.2 (br s, 2H, CH_2_), 1.4 (br s, 1H, CH), 1.7 (br s, 2H, CH_2_), 2.3 (t, 2H, CH_2_–N–CH_2_), 3.7 (t, 2H, CH_2_–N–CH_2_), 7.7 (dd, 1H, *J* = 8 Hz, CH–pyrimidine ring H_5_), 8.0 (dd, 1H, *J* = 12 Hz, AB–Ar–H), 8.4 (dd, 1H, *J* = 12 Hz, AB–Ar–H), 8.8 (s, 1H, CH–triazole ring), 9.0 (dd, 1H, *J* = 8 Hz, CH–pyrimidine ring H_6_); ^13^C-NMR (101 MHz, DMSO-*d_6_*) *δ*: 21.8, 29.7, 29.7, 33.4, 46.6, 46.6, 110.9, 128.0, 128.0, 131.1, 131.1, 134.1, 138.8, 146.2, 155.7, 156.1, 156.1; MS *m*/*z* (%): 357 (M^+^, 28.87); Anal. Calcd for C_17_H_19_N_5_O_2_S (357.43): C, 57.13; H, 5.36; N, 19.59; O; Found: C, 57.05; H, 5.27; N, 19.50%.

#### 3.1.11. 7-(4-((4-Methylpiperidin-1-yl)sulfonyl)phenyl)-2-phenylpyrazolo[1,5-*a*]pyramidine (**22**)

Pale brown crystals; yield 87%; m.*p*. 321–323 °C; IR (KBr) *υ* (cm^−1^): 3054 (Ar–CH), 2925 (Ali–CH), 1340, 1168 (SO_2_); ^1^H-NMR (400 MHz, DMSO-*d_6_*) *δ*: 0.9 (d, 3H, CH_3_), 1.2, (br s, 2H, CH_2_), 1.3 (br s, 1H, CH), 1.7 (br s, 2H, CH_2_), 2.3 (t, 2H, CH_2_–N–CH_2_), 3.7 (t, 2H, CH_2_–N–CH_2_), 7.5–7.9 (m, 6H, Ar–H and CH-pyrazole), 7.9 (dd, 1H, *J* = 8 Hz, CH–pyrimidine ring H_5_), 8.1 (dd, 1H, *J* = 8 Hz, AB–Ar–H), 8.3 (dd, 1H, *J* = 8 Hz, AB–Ar–H), 8.4 (dd, 1H, *J* = 8 Hz, CH–pyrimidine ring H_6_); MS *m*/*z* (%): 432 (M^+^, 3.10); Anal. Calcd for C_24_H_24_N_4_O_2_S (432.54): C, 66.64; H, 5.59; N, 12.95; O; Found: C, 66.55; H, 5.52; N, 12.87%.

#### 3.1.12. 7-(4-((4-Methylpiperidin-1-yl)sulfonyl)phenyl)-2-(methylthio)pyrazolo[1,5-*a*]pyrimidine-3-carbonitrile (**28**)

Pale yellow crystals; yield 85%; m.*p*. 182–184 °C; IR (KBr) *υ* (cm^−1^): 3092 (Ar–CH), 2930 (Ali–CH), 2223 (CN), 1328, 1166 (SO_2_); ^1^H-NMR (400 MHz, DMSO-*d_6_*) *δ*: 0.9 (d, 3H, CH_3_), 1.2 (br s, 2H, CH_2_), 1.4 (br s, 1H, CH), 1.7 (br s, 2H, CH_2_), 2.3 (t, 2H, CH_2_–N–CH_2_), 2.7 (s, 3H, CH_3_S), 3.7 (t, 2H, CH_2_–N–CH_2_), 7.6 (dd, 1H, *J* = 4 Hz, CH–pyrimidine ring H_5_), 8.0 (dd, 1H, *J* = 8 Hz, AB–Ar–H), 8.4 (dd, 1H, *J* = 8 Hz, AB–Ar–H), 8.9 (dd, 1H, *J* = 4 Hz, CH–pyrimidine ring H_6_); ^13^C-NMR (101 MHz, DMSO-*d_6_*) *δ*: 13.7, 21.8, 29.71, 29.7, 33.3, 46.5, 46.5, 80.4, 111.2, 113.1, 127.8, 127.9, 131.2, 131.3, 133.9, 139.0, 145.5, 152.7, 154.2, 158.1; MS *m*/*z* (%): 427 (M^+^, 100); Anal. Calcd for C_20_H_21_N_5_O_2_S_2_ (427.54): C, 56.19; H, 4.95; N, 16.38; O; Found: C, 56.11; H, 4.88; N, 16.29.

#### 3.1.13. General Procedure for Preparation of (**31a–c**)

A mixture of enaminone (4) (0.01 mol) and 4-(4-(hydroxyl, methyl or chloro)phenylazo)-1*H*-pyrazole-3,5-diamine (0.01 mol) in glacial acetic acid (30 mL) and fused sodium acetate (2 gm) was added. The subsequent mixture was refluxed for 6h, allowed at room temperature, and diluted with water (20 mL). The formed solid product was collected by filtration, washed with water, and recrystallized from methanol and benzene to furnish compounds **31a–c**. The physical and spectral data of molecules 31a-c were as illustrated:

#### 3.1.14. (*E*)-4-((2-Amino-7-(4-((4-methylpiperidin-1-yl)sulfonyl)phenyl)pyrazolo[1,5-*a*]pyrimidin-3-yl)diazenyl)phenol (**31a**)

Burgundy crystals; yield 89%; m.*p*. 276–277 °C; IR (KBr) *υ* (cm^−1^): 3438 (OH), 3264, 3190 (NH_2_), 3094 (Ar–CH), 2917 (Ali–CH), 1333, 1168 (SO_2_); ^1^H-NMR (400 MHz, DMSO-*d_6_*) *δ*: 0.9 (d, 3H, CH_3_), 1.2 (br s, 2H, CH_2_), 1.4 (br s, 1H, CH), 1.7 (br s, 2H, CH_2_), 2.3 (t, 2H, CH_2_–N–CH_2_), 3.7 (t, 2H, CH_2_–N–CH_2_), 6.9 (dd, 1H, *J* = 12 Hz, AB–Ar–H), 7.2 (s, 2H, NH_2_, exchangeable by D_2_O), 7.3 (dd, 1H, *J* = 4 Hz, CH–pyrimidine ring H_5_),7.7 (dd, 1H, *J* = 12 Hz, AB–Ar–H), 7.9 (dd, 1H, *J* = 8 Hz, AB–Ar–H), 8.3 (dd, 1H, *J* = 8 Hz, AB–Ar–H), 8.6 (dd, 1H, *J* = 4 Hz, CH–pyrimidine ring H_6_), 9.9 (s, 1H, OH, exchangeable by D_2_O); MS *m*/*z* (%): 491 (M^+^, 12.05); Anal. Calcd for C_24_H_25_N_7_O_3_S (491.57): C, 58.64; H, 5.13; N, 19.95; Found: C, 58.55; H, 5.04; N, 19.87%.

#### 3.1.15. (*E*)-7-(4-((4-Methylpiperidin-1-yl)sulfonyl)phenyl)-3-(*p*-tolyldiazenyl)pyrazolo[1,5-*a*]pyramidin-2-amine (**31b**)

Brown crystals; yield 86%; m.*p*. 305–307 °C; IR (KBr) *υ* (cm^−1^): 3264, 3190 (NH_2_), 3094 (Ar–CH), 2917 (Ali–CH), 1333, 1168 (SO_2_); ^1^H-NMR (400 MHz, DMSO-*d_6_*) *δ*: 0.9 (d, 3H, CH_3_), 1.2 (br s, 2H, CH_2_), 1.3 (br s, 1H, CH), 1.7 (br s, 2H, CH_2_), 2.3 (t, 2H, CH_2_–N–CH_2_), 2.4 (s, 3H, CH_3_), 3.7 (t, 2H, CH_2_–N–CH_2_), 7.3 (s, 2H, NH_2_, exchangeable by D_2_O), 7.7 (dd, 1H, *J* = 4 Hz, AB–Ar–H), 7.9 (dd, 1H, *J* = 4 Hz, AB–Ar–H), 8.3 (dd, 1H, *J* = 8 Hz, CH–pyrimidine ring H_5_), 8.6 (dd, 1H, *J* = 8 Hz, CH–pyrimidine ring H_6_); ^13^C NMR (101 MHz, DMSO-*d_6_*) *δ*: 21.3, 21.8, 29.7, 29.7, 33.4, 46.7, 46.6, 109.9, 114.9, 121.6, 127.9, 127.6, 130.1, 130.1, 131.0, 131.0, 135.0, 134.9, 138.2, 138.9, 143.9, 148.0, 151.1, 151.4, 152.4; MS *m*/*z* (%): 489.19 (M^+^, 12.05); Anal. Calcd for C_25_H_27_N_7_O_2_S (489.59): C, 61.33; H, 5.56; N, 20.03; Found: C, 61.26; H, 5.50; N, 19.98.

#### 3.1.16. (*E*)-3-((4-Chlorophenyl)diazenyl)-7-(4-((4-methylpiperidin-1-yl)sulfonyl)phenyl)pyrazolo[1,5-*a*]pyrimidin-2-amine (**31c**)

Orange crystals; yield 88%; m.*p*. 310–312 °C; IR (KBr) *υ* (cm^−1^): 3261, 3186 (NH_2_), 3095 (Ar–CH), 2921 (Ali–CH), 1330, 1169 (SO_2_); ^1^H-NMR (400 MHz, DMSO-*d_6_*) *δ*: 0.9 (d, 3H, CH_3_), 1.2 (br s, 2H, CH_2_), 1.4 (br s, 1H, CH), 1.7 (br s, 2H, CH_2_), 2.3, (t, 2H, CH_2_-N-CH_2_), 3.7 (t, 2H, CH_2_-N-CH_2_), 7.3 (s, 2H, NH_2_, exchangeable by D_2_O), 7.4 (dd, 1H, *J* = 4 Hz, AB–Ar–H), 7.6 (dd, 1H, *J* = 4 Hz, AB–Ar–H), 7.9 (dd, 1H, *J* = 8 Hz, AB–Ar–H), 8.0 (dd, 1H, J = 8 Hz, AB–Ar–H), 8.3 (dd, 1H, *J* = 8 Hz, CH–pyrimidine ring H_5_), 8.7 (dd, 1H, *J* = 8 Hz, CH–pyrimidine ring H_6_); MS *m*/*z* (%): 509 (M^+^, 35.44); Anal. Calcd for C_24_H_24_ClN_7_O_2_S (509.50): C, 56.52; H, 4.74; N, 19.22; O; Found: C, 56.53; H, 4.67; N, 19.14.

#### 3.1.17. 5-(4-((4-Methylpiperidin-1-yl)sulfonyl)phenyl)-2-thioxo-2,3-dihydropyrido[2–*d*]pyrimidin-4(1*H*)-one (**33**)

A mixture of enaminone (**4**) (0.01 mol) and 6-amino-2-thioxo-*2,3*-dihydro-1*H*-pyrimidin-4-one (0.01 mol) in glacial acetic acid (30 mL) was refluxed for 3h. The solvent was removed by distillation under reduced pressure, which was, subsequently, left to cool. The solid precipitate was collected by filtration and recrystallized from methanol/benzene to yield derivative 33. The physical and spectral data of compounds **33** was as demonstrated:

Pale yellow crystals; yield 88%; m.*p*. 289–291 °C; IR (KBr) *υ* (cm^−1^): 3394 (NH), 3062 (Ar–CH), 2925 (Ali–CH) 1687 (C=O), 1341, 1162 (SO_2_); ^1^H-NMR (400 MHz, DMSO-*d_6_*) *δ*: 0.9 (d, 3H, CH_3_), 1.2 (br s, 2H, CH_2_), 1.3 (br s, 1H, CH), 1.7 (br s, 2H, CH_2_), 2.3 (t, 2H, CH_2_–N–CH_2_), 3.7 (t, 2H, CH_2_–N–CH_2_), 7.9 (dd, 2H, *J* = 8 Hz, AB–Ar–H), 8.0 (dd, 2H, *J* = 8 Hz, AB–Ar–H), 8.4 (dd, 1H, *J* = 4 Hz, CH-pyridine ring H_6_), 8.4 (dd, 1H, *J* = 4 Hz, CH-pyridine ring H_7_), 12.7, 13.2 (2s, 2H, 2NH); ^13^C-NMR (101 MHz, DMSO-*d_6_*) *δ*: 21.8, 29.8, 29.9, 33.3, 46.7, 46.6, 112.1, 118.0, 128.5, 128.6, 128.7, 128.8, 137.7, 138.2, 141.2, 152.1, 159.4, 160.0, 176.7; MS *m/z* (%): 416 (M^+^, 13.65); Anal. Calcd for C_19_H_20_N_4_O_3_S_2_ (416.51): C, 54.79; H, 4.84; N, 13.45; O; Found: C, 54.71; H, 4.77; N, 13.37%.

### 3.2. Molecular Modeling

The in-silico experiments were conducted using MOE software, Chemical Computing Group’s Molecular Operating Environment, 2014.09 release, installed on a SAMSUNG workstation with Intel(R) Core (TM) i7–6500U CPU @ 2.5 GHz processor and 12.0 GB RAM.

### 3.3. Protein Structure Preparation

The X-ray crystal structure of the ERK1 (PBD code: 6GDM) was acquired from the Protein Data Bank (www.rcsb.com). The Structure Preparation application of MOE was implemented to prepare the kinase domain, where structural issues as alternates, termini, hydrogen count, and incorrect charges have been addressed and corrected. The Protonate3D was implemented to identify residues with possible rotamers, protomers, or tautomeric states. Finally, energy minimization was applied, exploiting an MMFF94x forcefield with default parameters.

### 3.4. Ligand Structure Preparation

The 2D structures of the molecules were sketched, employing ChemDraw User Interface version 15.0, and were saved as MDL Molfile. The structures were then imported on the MOE interface, and the 3D structures of the molecules were generated for conformational search. The geometry optimization and energy minimization were then implemented for the generated 3D structures.

### 3.5. Molecular Docking

The prepared X-ray crystal structure ERK1 kinase domain and the 3D optimized structures of the synthesized molecules were employed to the subsequent docking experiments. The Rigid Receptor docking protocol was implemented for docking studies, using the Triangle Matcher for Placement and the London dG for Rescoring and Force field for Refinement.

### 3.6. Biological Screening

#### 3.6.1. Cell Culture

The tumor cell lines: mammary gland breast cancer cell line (MCF-7), human colon carcinoma (HCT-116), and hepatocellular carcinoma (HepG-2), were attained from the American Type Culture Collection (ATCC, Rockville, MD, USA). The cells were grown on an RPMI-1640 medium, supplemented with a 10% inactivated fetal calf serum and 50 µg/mL gentamycin. The cells were maintained at 37 °C in a humidified atmosphere with 5% CO_2_ and were subculture two to three times a week.

#### 3.6.2. Evaluation of Anti-Proliferative Activity

The cytotoxicity was appraised, exercising the standard sulphorhodamine B (SRB) assay, as reported previously [61].

### 3.7. Cell Cycle Analysis

The cell cycle distribution was assessed, using the Propidium Iodide (PI) Flow Cytometry Kit (ab139418, Abcam, Cambridge, UK), followed by the flow cytometric analysis. Briefly, the 5 × 10^4^ cells were seeded in a 60 mm culture dish and incubated for 24 h to form a cell monolayer. The cells were cultured for an additional 24 h. in the absence of DMSO (negative control) or in the presence of Vinblastine and Doxorubicin (positive standard controls) or the synthesized compounds **5**, **9**, **10b**, **22**, and **28** at their corresponding IC_50_ values in the proliferation assay. The adherent cells were trypsinized, washed with PBS, and fixed in 100% ice-cold ethanol at 4 °C for at least 2 h. The ethanol was removed, and the cells were washed with PBS before incubating with 200 μL 1X Propidium Iodide (PI)+RNase Staining Solution for 30 min at room temperature in dark. The DNA content was determined by a FACS Calibur flow cytometer (BD Biosciences, Franklin Lakes, NJ, USA). Finally, the cell cycle phase distribution was analyzed, using the Cell Quest Pro software (BD Biosciences), which displays the collected propidium iodide fluorescence intensity on FL2.

### 3.8. Flow Cytometry by Annexin V-FITC

The cell apoptosis was evaluated by the Annexin V-FITC/PI double staining apoptosis detection kit (K101, BioVision, Milpitas, CA, USA), using a flow cytometer. The cell culture was prepared, as reported for the cell cycle analysis assay with or without the tested compounds. The staining procedure was performed, following the manufacturer’s instructions. A minimum of 10,000 cells per sample were acquired. The Annexin V-FITC binding (FL1) and PI (FL2) were analyzed, employing the Cell Quest Pro software (BD Biosciences).

### 3.9. Statistical Analysis

All the biological data were expressed as means ± standard deviation (SD) of at least three independent experiments. The statistical analysis was performed by the GraphPad Prism 5.01 (GraphPad software, San Diego, CA, USA). The data were analyzed, using ANOVA, followed by the Tukey’s *post hoc* test. The statistical significance is indicated as * *p* ≤ 0.05, ** *p* ≤ 0.01, and *** *p* ≤ 0.001.

## 4. Conclusions

Targeting oncogenic protein kinases is a robust molecular therapeutic strategy to control human malignancies. The interference with a Mitogen-Activated Protein (MAP) kinase signaling pathway by small molecule inhibitors has a distinguished and remarkable impact on hampering cancerous cell proliferation, the stimulation of cell cycle arrest, and apoptosis. The rational synthesis of ERK inhibitors based on a molecular extension tactic of a small fragment bounded at the kinase domain is a valid scheme to enhance the overall binding affinity and, thus, improving the biological potency. This report establishes an efficacious route in developing a novel class of pyrimidine molecules bearing sulfonamide moieties. The structural identities of the new derivatives were validated and their cytotoxic behavior were explored. Alongside their in vitro assay, the molecular docking and cell cycle analysis revealed that the sulfonamides, linked to triazolo[4,3-*a*]pyrimidine, pyrazolo[1,5-*a*]pyrimidine and pyrido[2–*d*]pyrimidine, are excellent scaffolds for constructing small molecules ERK inhibitors.

## Figures and Tables

**Table 1:** The IC_50_ values of the target compounds against the MCF-7, HCT-116, and HepG-2 lines.

Compound		IC_50_ (µM) ^a^	
	MCF-7	HCT-116	HepG-2
**3**	100 ± 0.12	85.4 ± 0.03	84.52 ± 0.12
**4**	27.05 ± 0.35	25.44 ± 0.16	26.27 ± 0.5
**5**	9.96 ± 0.2	6.92 ± 0.11	2.56 ± 0.14
**6**	22.28 ± 0.13	18.56 ± 0.12	16.78 ± 0.11
**9**	10.43 ± 0.12	10.14 ± 0.05	4.36 ± 0.02
**10a**	58.97 ± 0.3	48.48 ± 0.6	36.94 ± 0.1
**10b**	9.89 ± 0.4	7.09 ± 0.1	5.72 ± 0.12
**16**	100 ± 0.13	97.22 ± 0.12	97.05 ± 0.11
**22**	7.84 ± 0.02	7.28 ± 0.1	2.96 ± 0.12
**28**	8.19 ± 0.03	5.38 ± 0.2	4.21 ± 0.01
**31a**	14.69 ± 0.06	10.37 ± 0.18	9.36 ± 0.16
**31b**	45.75 ± 0.1	42.08 ± 0.04	35.23 ± 0.02
**31c**	57.61 ± 0.11	42.58 ± 0.13	31.19 ± 0.6
**33**	15.62 ± 0.12	14.07 ± 0.13	14.41 ± 0.11
Vinblastine	5.83 ± 0.13	3.2 ± 0.09	7.35 ± 0.42
Doxorubicin	8.19 ± 0.72	6.74 ± 0.68	7.52 ± 0.51

^a^ IC_50_ values expressed in µM as the mean values of triplicate wells from at least three experiments and are reported as the mean ± standard error.
